# Looking beyond the numbers: quality assurance procedures in the Global Network for Women’s and Children’s Health Research Maternal Newborn Health Registry

**DOI:** 10.1186/s12978-020-01009-3

**Published:** 2020-11-30

**Authors:** Ana Garces, Emily MacGuire, Holly L. Franklin, Norma Alfaro, Gustavo Arroyo, Lester Figueroa, Shivaprasad S. Goudar, Sarah Saleem, Fabian Esamai, Archana Patel, Elwyn Chomba, Antoinette Tshefu, Rashidul Haque, Jacquelyn K. Patterson, Edward A. Liechty, Richard J. Derman, Waldemar A. Carlo, William Petri, Marion Elizabeth M. Koso-ThomasMcClure, Robert L. Goldenberg, Patricia Hibberd, Nancy F. Krebs

**Affiliations:** 1 Instituto de Nutrición de Centroamérica y Panamá, Guatemala, Guatemala; 2 RTI International, Durham, NC USA; 3 KLE Academy Higher Education and Research, J N Medical College, Belagavi, Karnataka India; 4 Aga Khan University, Karachi, Pakistan; 5 Moi University School of Medicine, Eldoret, Kenya; 6 Lata Medical Research Foundation, Nagpur, India; 7 University Teaching Hospital, Lusaka, Zambia; 8 University of Kinshasa School of Public Health, Kinshasa, Democratic Republic of the Congo; 9 International Centre for Diarrhoeal Disease Research, Bangladesh (icddr,b), Dhaka, Bangladesh; 10 University of North Carolina at Chapel Hill, Chapel Hill, NC USA; 11 Indiana School of Medicine, University of Indiana, Indianapolis, IN USA; 12 Thomas Jefferson University, Philadelphia, USA; 13 University of Alabama at Birmingham, Birmingham, AL USA; 14 University of Virginia, Charlottesville, VA USA; 15 Eunice Kennedy Shriver National Institute of Child Health and Human Development, Bethesda, MD USA; 16 Department of Obstetrics and Gynecology, Columbia University School of Medicine, New York, NY USA; 17 Boston University School of Public Health, Boston, MA USA; 18 University of Colorado School of Medicine, Denver, CO USA

**Keywords:** Quality assurance, Training for research studies, Adult learning, Quality control, Public health training, Global network

## Abstract

**Background:**

Quality assurance (QA) is a process that should be an integral part of research to protect the rights and safety of study participants and to reduce the likelihood that the results are affected by bias in data collection. Most QA plans include processes related to study preparation and regulatory compliance, data collection, data analysis and publication of study results. However, little detailed information is available on the specific procedures associated with QA processes to ensure high-quality data in multi-site studies.

**Methods:**

The Global Network for Women’s and Children’s Health Maternal Newborn Health Registy (MNHR) is a prospective population-based registry of pregnancies and deliveries that is carried out in 8 international sites. Since its inception, QA procedures have been utilized to ensure the quality of the data. More recently, a training and certification process was developed to ensure that standardized, scientifically accurate clinical definitions are used consistently across sites. Staff complete a web-based training module that reviews the MNHR study protocol, study forms and clinical definitions developed by MNHR investigators and are certified through a multiple choice examination prior to initiating study activities and every six months thereafter. A standardized procedure for supervision and evaluation of field staff is carried out to ensure that research activites are conducted according to the protocol across all the MNHR sites.

**Conclusions:**

We developed standardized QA processes for training, certification and supervision of the MNHR, a multisite research registry. It is expected that these activities, together with ongoing QA processes, will help to further optimize data quality for this protocol.

## Plain english summary

All research studies should have quality assurance, as this protects the rights and safety of study participants. It also improves the quality of data collection and improves the likelihood of obtaining true results. Most quality assurance plans cover a variety of topics, including the preparation prior to the study, ethics issues, analysis of data and publication of study results. There is limited information on the procedures that different studies use to ensure data of high quality.

The procedures that we report here were done as part of the The Global Network for Women’s and Children’s Health Maternal Newborn Health Registy (MNHR), registry of pregnancies and deliveries that is carried out in 8 international sites. The MNHR has adoptes quality assurance procedures since its beginning, to ensure the quality of the data. More recently we developed a training and certification process to ensure that the same clinical definitions be used across these eight sites. The study staff are trained on these defitions with a web-based training module which covers the study protocol, study forms and clinical definitions. They are later certified before initiating study activities and every six months thereafter. The MNHR also carries out a procedure for supervision and evaluation of field staff to ensure that research activites are conducted according to the protocol across all sites. We expect that all of these activities will help to further optimize data quality for this protocol.

## Background

Quality assurance (QA) is a process that should be carried out throughout all phases of research to protect the rights and safety of study participants, to improve consistency in data and to reduce the likelihood that trial results are affected by bias [1, 2]. QA seeks to ensure that studies comply with research standards, to detect problems early through routine monitoring and to correct issues through prompt and effective action [3]. Such processes should be considered a standard part of all research activities.

Most QA plans include processes related to study preparation and regulatory compliance, data collection, data analyses and publication of study results [1]. Additionally, multi-site studies generally include common variables, data collection methodologies and standardized protocols. However, although there is consensus on the importance of data quality for research, little detailed information is available on the specific procedures and best practices for QA processes [4]. One research study from India recently published a data quality assurance protocol, which focused on tools to ensure the accuracy, reliability, timeliness, completeness, precision, and integrity of the data [5]. The investigators found that the tools helped increase accuracy of data collection throughout the research project. With the increasing global emphasis on harmonization and data sharing in research, ensuring not only high quality of data but also comparability of data across diverse settings is critical to accurate interpretation of findings [6, 7].

The Maternal Newborn Health Registry (MNHR) is a prospective, population-based registry of pregnancies and deliveries conducted under the auspices of the Global Network for Women’s and Children’s Health Research (GN), a multi-country research network. The MNHR enrolls approximately 60,000 pregnant women each year and follows them from early pregnancy through the postnatal period [8, 9]. Briefly, its primary purpose is to quantify and analyze trends in pregnancy outcomes over time across the GN research sites. It also serves as a data collection tool for capturing pregnancies, perinatal and neonatal outcomes for individual studies [10, 11] and provides data to plan future GN studies. Additionally, MNHR data are frequently provided to local health officials who use it to inform and improve clinical care.

Because the MNHR operates in multiple sites in diverse low and middle-income countries (LMIC) and collects sensitive data on a large scale, QA has been particularly integral to ensuring quality data collection since the Registry’s inception in 2008. At the start of the MNHR, the investigators determined the critical data to collect, developed common definitions based on the WHO criteria, and also defined common methods to collect the data. In addition, the MNHR introduced a process of ongoing data quality monitoring, including metrics to assess missingness and accuracy, which was conducted both with local research teams and centrally, with a process for rapid feedback and resolution of data issues (Table 1) [12].

**Table 1:** Elements of the quality assurance plan for the Global Network’s Maternal and Newborn Health Registry

Study preparation and regulatory compliance	Institutional Review Board and Ethical Review Committee approved protocol, consents and recruitment materials Curriculum vitaes and documentation of qualifications of investigators Case report forms Staff training^a^
Data collection	Standard operating procedures Manual of operations Monitoring plan Supervisory visits^a^
Data analysis	Monthly monitoring reports Quarterly monitoring metrics Data Monitoring Committee

^a^Recently implemented QA procedures

In 2017, in an effort to continously improve the quality of data in the MNHR and to further ensure comparability across diverse sites, we identified a need to standardize the clinical data collected by field staff across all sites. To address this gap, we developed additional procedures for training, certification and supervision of all staff within the MNHR. In this this paper, we describe the development of tools to support the standardization and enhanced QA of data collection including a web-based standardized training and certification procedure and an evaluation tool for consistent supervision of field staff at all GN sites. Additionally, we describe our proposed approach to evaluate the feasibility and effectiveness of these newly-implemented procedures.

## Methods

### Setting and background

The GN MNHR was established in research sites based in Argentina, Guatemala, India (two sites), Kenya, Pakistan and Zambia [5]. A site in the the Democratic Republic of the Congo (DRC) replaced the site in Argentina in 2013 and a Bangladeshi site was added in 2019. Investigators from each participating site and a partner United States-based institution, the NICHD and the Data Coordinating Center (DCC) based at RTI International, comprise the GN MNHR subcommittee that oversees all aspects of protocol design, study implementation, data analyses and publications. The GN MNHR is conducted under the auspices of the GN, a multi-disciplinary research network, which is supported by research grants from the National Institute of Child Health and Human Development (NICHD).

**Fig. 1:**
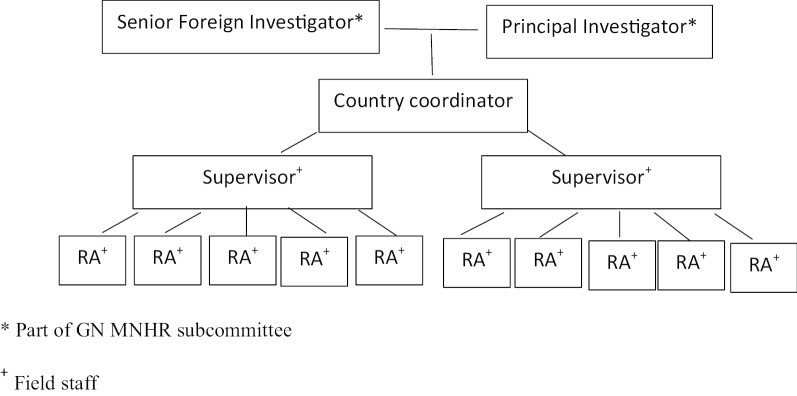
Maternal Newborn Health Registry study organization at site

For each site, the Principal Investigator (PI; based at a United States university), and the Senior Foreign Investigator (SFI; based at an international research site) are collectively responsible for ensuring the overall quality of the site’s data. A country coordinator (CC) provides study oversight in the field; one or more study supervisors train and supervise registry administrators (RAs). RAs have a variety of backgrounds, and have various types and levels of professional training (i.e., midwives, community health workers, physicians, nurses, medical technologists, health assistants, and social workers); however, a minimal requirement is community health worker level experience. They work with a wide range of public and private providers and collect data in multiple settings including participants´ homes and health institutions (Fig. 1). The RAs are paid study staff with participation in ongoing training considered part of their research responsibilities.

### QA procedures in place since the inception of the MNHR

#### Study preparation and regulatory compliance

In preparation for implementation of the MNHR, each of the GN study sites obtained approval of the study protocol, consent and case report forms (CRFs) from their respective Institutional Review Board both in the US and in-country. All research staff were certified in the Protection of Human Subjects and Good Clinical Practices. In accordance with NICHD policy, the study protocol, manual of procedures (MOP) and CRFs are publicly available through the GN website (https://gn.rti.org/); de-identified study data is available for secondary analyses through the NICHD Data and Specimen (N-DASH) hub (https://dash.nichd.nih.gov/).

#### Data collection

Standard operating procedures for data collection are detailed in the MNHR MOP which has been in place since study inception; it is reviewed annually and updated as needed. A question by question (QxQ) document defines each study question and is updated as needed; a policy document includes technical memos that describe new study procedures and outlines the addition or elimination of variables.

#### Data processing, analysis and publication

All MNHR data are entered locally into a computer-based data management system (DMS) that incorporates inter and intra-form data quality checks. The DCC produces monthly monitoring reports which detail site and cluster specific metrics to identify issues related to completeness of data collection and to review changes in outcomes measure quality improvements over time. Specifically, the reports focus on critical data, as defined by the central working group to ensure the completeness and accuracy of those data elements. Each site reviews these monthly monitoring reports and participates in quarterly monitoring calls [8]. Study data are reviewed annually by a Data Monitoring Committee. A publication management system outlines specific procedures for publication of study results.

### QA procedures

In an effort to continuously improve the quality of our data collection, we developed a standardized training and certification process for RAs as well as standardized supervisory procedures.

#### Training and certification

To improve data collection, including the use of standardized clinical definitions, we developed a web-based, interactive training module for MNHR RAs.

### Development and pilot evaluation

Prior to developing the module, we developed standardized definitions for all clinical data collected in the MNHR through an iterative process. First, we reviewed the MNHR CRFs and identified all clinical data fields (Table 2). We then conducted extensive searches for definitions of these terms using a variety of sources including the WHO and United Nations Population Fund websites as well as technical documents, clinical textbooks and peer reviewed journals. Based on these definitions, we compiled draft definitions for the MNHR that were reviewed by the GN MNHR subcommittee and study site investigators, including obstetric and pediatric specialists in the GN, for scientific accuracy. The subcommittee further refined these definitions during an in-person discussion to ensure they reflected the diagnostic capability of current healthcare at the MNHR research sites. For example, the final MNHR definition for malaria does not require confirmatory laboratory testing, as GN sites with high malaria prevalence diagnose the disease clinically without routinely performing confirmatory laboratory testing. Similarly, the definition of neonatal sepsis does not require confirmation with blood culture. Lastly, a medical editor reviewed the final draft definitions, also ensuring the literacy level was appropriate for the level of medical training required of MNHR RAs. As the next step, the modules were pilot tested with a sample of learners from the sites. These staff provided feedback on the definitions as well as the use of pictorial images.

**Table 2:** Clinical processes of care and health outcomes collected on Maternal and Newborn Health Registry forms, 2019

Pregnancy	Delivery	Postpartum and neonatal
*Outcomes* Ectopic pregnancy Miscarriage or spontaneous abortion Induced abortion Abortive related outcome Malaria Syphilis Hypertensive disease/preeclampsia/eclampsia^a^ Fetal or vaginal odor Other maternal infection *Processes of care* Dilation and curetage or suction	*Outcomes* Transverse lie Oblique lie Breech lie Severe antepartum hemorrhage^a^ Obstructed/prolonged labor, failure to progress Obstetric fistula Severe postpartum hemorrhage^a^ Severe infection Acute inversion of the uterus Maternal death^a^ Signs of maceration Stroke/loss of consciousness/paralysis Stillbirth^a^ Birth trauma/difficult delivery Cord complication Major malformation at birth *Processes of care* Unplanned hospitalization Antibiotics Corticosteroids Oxytocin Misoprostol Magnesium Sulphate Induction of labor Episiotomy Blood transfusion Hysterectomy Forceps/vacuum extraction delivery	*Outcomes* Congenital anomalies Abdominal wall defect Neural tube defect Breathing difficulties Prematurity Breathed weekly or did not cry at birth Fits/seizures of the neonate fever or low temperature chest X ray or clinical finding of pneumonia Pus draining from umbilical stump Asphyxia (neonate) Sepsis (neonate) Accident/assault/trauma/suicide Diabetes Severe anemia Severe jaundice Infection Seizures Signs of fetal distress *Processes of care* Required resuscitation at birth Continued positive airway pressure Oxygen Mechanical ventilation Medicinal eye care Medicinal cord care

^a^Common to more than one period

### Implementation of web-based QA

Using these standardized clinical definitions, study protocol and MOP, the Instituto de Nutrición de Centro América y Panamá (INCAP) developed a web-based training module with *Storyline 360 *(https://360.articulate.com) software, which supports the development of interactive courses for all types of computers and devices. This training module was designed using andragogic learning principles for adults, facilitating knowledge acquisition by linking new concepts to previous experiences and prior knowledge [13]. The course contextualizes the learning process to the MNHR setting, so that learners can establish an immediate link between theory (such as clinical definitions) and its practical application. The module covers the study objectives, protocol, and CRFs including instructions for use and standardized definitions for all clinical data fields.

This web-based module is available to all sites through the GN webpage, which can be accessed through study computers or tablets as well as in an off-line mode. The module engages learners through key information and communication technology. It was developed in English and translated into Spanish and French, with subtitles appearing throughout the course. If additional languages are required, the local staff provide translations. Clinical definitions are communicated with multi-media, using text, audio, and an image illustrating the concept. Brief quizzes, which require matching the definition to the concept after review of every three clinical terms, are interspersed throughout the module to help learners assess their comprehension. Finally, the course highlights achievements of the learner as he/she completes each section. Techincal assistance is provided centrally by RTI staff, as needed, to complete the modules.

Following the training module, RAs complete a multiple-choice certification exam (available to all sites through the GN webpage or via an electronic copy on a storage device). Initial certification, and recertification, require a minimum score of 80% on this exam. If an RA does not attain this score, he/she receives additional training from the country coordinator and repeats the training module before re-taking the exam. All RAs recertify by obtaining a passing grade on the certification exam every six months. Additionally, RAs have access to the module for additional training whenever necessary. Altogether, the training and certification process takes approximately 8–10 h for each staff to complete.

#### Supervisory visits

To improve data collection and study implementation, the subcommittee also standardized supervisory procedures of RAs across sites.

First, each site submitted a description of their site-specific supervisory evaluation process, including any corresponding forms. Based on these descriptions and the recommendations for implementation of QA processes, the subcommittee developed a standardized supervisory procedure with corresponding CRF for usage by all sites in the MNHR. This CRF assesses general activities of the RA, communication with other health providers and a field visit, including a key variable check. Country coodinators piloted the supervisory process and forms in each site. They informally found the proposed procedures and frequency of supervision to be feasible. Additionally, they gave feedback on the data selected for variable checks such as using maternal height instead of weight since the latter can fluctuate between when an RA records the data and a supervisor confirms it. Final variables were selected based on their likelihood of being easily recalled by the mother and readily obtained during a supervisory home visit, and included such items as maternal height, delivery location, and mode of delivery.

The final, standardized supervisory procedure occurs as follows:

Annually, all RAs undergo two supervisory visits consisting of three parts, which are recorded on a study form. The supervisor arrives in the community where the RA is scheduled and corroborates that he/she is prepared with consents, study forms and equipment. The supervisor visits community level or ministry of health staff to corroborate that the RA interfaces regularly and appropriately with them. Finally, the supervisor visits two randomly selected MNHR participants to corroborate data in the DMS for specific key variables. Supervisors review the findings of these supervision processes with each RA. If necessary, RAs are provided additional training targeting non-compliance with study procedures or errors in data collection through the training module or direct coaching by supervisors.

#### Evaluation plan of recently implemented QA procedures

Country coordinators ensure that all RAs complete training and certification procedures and report on these processes to the DCC, which keeps a log of these data. Data collected through the supervisory process are entered into the DMS and analyzed by the DCC. Results of both are discussed on periodic site calls.

To evaluate the impact of these QA methods, the subcommittee will review the training and certification procedure for feasibility (such as the number of RAs trained and average time for course completion) and effectiveness (such as certification exam scores and first-time pass rate). Through data collected during our supervisory procedure, we will evaluate RA fidelity to the study protocol and quality of data collected (such as percent of data elements in the DMS that is congruent with participant-reported data for each key variable). We will track congruency of all key variables on the CRF over time to determine whether our QA training is successful at improving the quality of the data. Additionally, the central QA team reviews the content and updates, as needed, on a bi-annual basis.

## Discussion

QA is a necessary part of conducting research; best practices recommend that it includes measures to prevent, detect and correct errors from the beginning of data collection through the publication of study results. QA also helps ensure that data are accurate and collected using common methods across sites. This is particularly challenging in multi-site, large-scale studies such as the MNHR. Multi-site studies need to ensure standardization of definitions across diverse settings to help ensure generalizability of findings. In addition to the standardized definitions and procedures, other studies have emphasized the importance of leveraging local capacity with central technical support, similar to the model of the GN [14, 15]. Additionally, as with the GN, the use of ongoing data metrics in routine monitoring reports have been shown to improve data quality [5, 16].

The MNHR includes participants in eight diverse LMIC settings, enrolling approximately sixty thousand participants annually. Since its inception ten years ago, we designed QA procedures for data collection, entry, editing and transmission. In 2012, we added metrics and standardized reports to facilitate regular identification of site-specific implementation issues. These QA procedures have supported the successful enrollment of 704,265 participants with over 95% followed from pregnancy through delivery [9].

Building upon the existing platform of common definitions and ongoing data monitoring, in an effort to continuously improve the quality of our data in 2017 we implemented standardized training and certification as well as supervisory procedures with a number of notable strengths. Our training and certification procedure includes newly-developed, standardized definitions for all clinical data collected in the MNHR. The web-based training module utilizes andragogic learning principles and capitalizes on information and communication technology to engage learners. Recertification of RAs ensures that initial proficiency in study procedures is maintained. The supervisory procedure facilitates the detection of non-compliance with study procedures as well as errors in data collection, reporting and entry. All GN sites have successfully implemented these training, certification and supervisory procedures, and given the relatively low additional burden in terms of time and other resources, have found that they are feasible to continue. We thus view these changes in procedures to be strengths that will enhance the utility of this registry for its multiple intended purposes.

Despite these strengths, there are limitations to our newly-implemented QA procedures. The MNHR data are collected via medical record abstraction and interview of participants. While we developed standardized clinical definitions to support accurate data collection, these definitions do not ensure accurate ascertainment and documentation of clinical diagnoses by health care providers, nor do they ensure accurate maternal recall. Similarly, while we attempted to verify variables most likely to be recalled by the mother during supervisory visits, maternal recall is an imperfect ‘gold standard’.

In this manuscript, we have described a comprehensive QA procedure that may serve as a model for other multi-site large-scale studies. Through routine MNHR review processes, we identified an opportunity to improve training and oversight with the goal of assuring strong adherence to a protocol implemented in diverse low resource settings. On-going data collection regarding these processes will allow us to evaluate their feasibility and effectiveness, and to determine critical elements for maintaining high quality data for this and other similar registry—based protocols.

## Conclusion

In conclusion, especially for large, multi-site clinical research studies in LMICs, the ability to harmonize data across diverse settings presents challenges, limiting the ability to compare site results. Providing a standardized training across the sites together with reinforced supervision and oversight that is centrally monitored has proven useful in improving quality of data. The approaches used to facilite QA across the MNHR have applicability across other multi-site research studies, which have particular challenges in ensuring common methodologies and interpretation of data.

## Data Availability

Data from the study will be available at the NICHD data repository (N-DASH): https://dash.nichd.nih.gov/.

## Abbreviations

QA: Quality assurance
MNHR: Maternal Newborn Health Registy
GN: Global Network for Women’s and Children’s Health Research
NICHD: Eunice Kennedy Shriver National Institute of Child Health and Human Development
LMIC: Low and middle-income countries
DRC: Democratic Republic of the Congo
DCC: Data coordinating center
SFI: Senior foreign investigator
CC: Country coordinator
RAs: Registry administrators
CRFs: Case report forms
MOP: Manual of procedures
N-DASH: NICHD Data and Specimen
QxQ: Question by question
DMS: Data management system
INCAP: Instituto de Nutrición de Centro América y Panamá
